# Prediction of hemophilia A severity using a small-input machine-learning framework

**DOI:** 10.1038/s41540-021-00183-9

**Published:** 2021-05-25

**Authors:** Tiago J. S. Lopes, Ricardo Rios, Tatiane Nogueira, Rodrigo F. Mello

**Affiliations:** 1 Department of Reproductive Biology, National Center for Child Health and Development Research Institute, Tokyo, Japan; 2 Department of Computer Science, Federal University of Bahia, Salvador, Brazil; 3 Institute of Mathematics and Computer Science, University of São Paulo, São Carlos, Brazil; 4 Present Address: Itaú Unibanco, Av. Eng. Armando de Arruda Pereira, São Paulo, Brazil

**Keywords:** Protein engineering, Structural biology, Biochemical networks

## Abstract

Hemophilia A is a relatively rare hereditary coagulation disorder caused by a defective F8 gene resulting in a dysfunctional Factor VIII protein (FVIII). This condition impairs the coagulation cascade, and if left untreated, it causes permanent joint damage and poses a risk of fatal intracranial hemorrhage in case of traumatic events. To develop prophylactic therapies with longer half-lives and that do not trigger the development of inhibitory antibodies, it is essential to have a deep understanding of the structure of the FVIII protein. In this study, we explored alternative ways of representing the FVIII protein structure and designed a machine-learning framework to improve the understanding of the relationship between the protein structure and the disease severity. We verified a close agreement between in silico, in vitro and clinical data. Finally, we predicted the severity of all possible mutations in the FVIII structure – including those not yet reported in the medical literature. We identified several hotspots in the FVIII structure where mutations are likely to induce detrimental effects to its activity. The combination of protein structure analysis and machine learning is a powerful approach to predict and understand the effects of mutations on the disease outcome.

## Introduction

Hemophilia A (HA) is an X-linked heritable disease affecting approximately 1 in every 5000–10,000 live male births^1^. People with hemophilia A (PwHA) have endogenous defective copies of the coagulation factor VIII gene, which in turn dramatically affects the blood coagulation cascade. As a result, depending on the type of mutation on the F8 gene, disease symptoms may vary from mild (when PwHA only experience rare bleeding episodes, clotting activity level 5–40%), to moderate (where these episodes are more frequent, clotting activity 1–5%), and severe (when there is a permanent risk of bleeding complications and chronic joint damage^2^, clotting activity <1%).

Although it is a relatively rare disorder, the coagulation pathway is well-characterized and treatment options are improving since the 1950s, evolving from blood-derived FVIII concentrates^2^ to recombinant proteins^3^ and a recent monoclonal antibody^4^. These so-called replacement therapies aim to replace the defective FVIII protein through constant supplementation of one of these products.

Although the life expectancy and the quality of life of PwHA improved considerably in the last decades^2,5^, current treatment options still have issues to be addressed; for instance, it is necessary to improve the half-life of recombinant FVIII proteins (currently ~12–19 h, considering the standard and extended half-life products), as well as its immunogenic profiles to avoid the development of neutralizing antibodies^3^. Finally, it is also important to develop recombinant proteins suitable for both prophylaxis and treatment of severe bleeding episodes^6^.

To overcome these drawbacks, a thorough understanding of the FVIII protein structure is essential. Reported mutations include deletions and inversions of large parts of its encoding gene, as well as single-nucleotide polymorphisms that do not change the reading frame, but replace an amino acid and generate a defective protein. Using gene information and protein structure properties, previous studies revealed fundamental aspects of single amino acid changes and their relation to severe or mild forms of HA^7–12^. However, the lack of strict data curation and the analysis of each property in isolation prevented these methods from predicting and mechanistically understanding the occurrence of mild, moderate, and severe phenotypes.

**Fig. 1:**
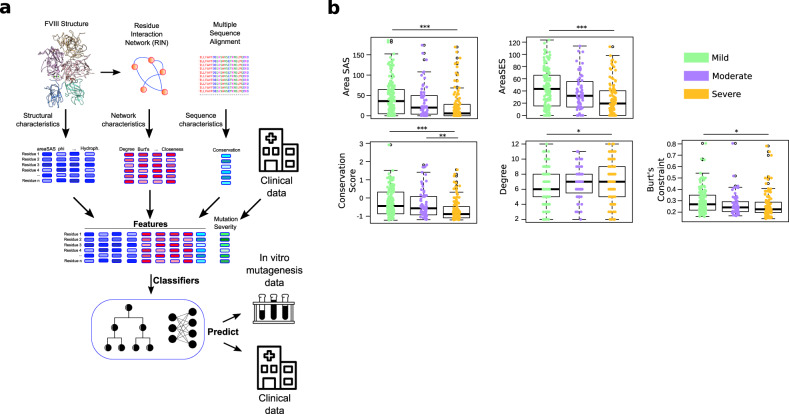
Structural and nonstructural properties of the FVIII protein. **a** To assemble the dataset we combined structural properties from the FVIII protein structure, from a multiple sequence alignment (i.e., the conservation of residues), and from a residue interaction network (RIN). In a RIN, each amino acid is a node, and two nodes are connected if the two residues are at close proximity in the structure (i.e., if their main- or side-chains are less than ~5 Å from each other). The FVIII RIN had 1336 nodes and 4074 edges. **b** Distribution of values of the variables related to the HA severity. Depicted is the solvent-accessible (areaSAS) and the solvent-excluded (areaSES) surface areas, the conservation of the FVIII residues (conservation score—smaller values indicate higher conservation). The degree is the number of connections that a node has, and Burt’s constraint^21^ quantifies the strategic location of a node between groups that would not be connected otherwise. The boxplots depict the median (centerline), the first and third quartiles (lower- and upper-bounds), and 1.5 times the inter-quartile range (lower- and upper whiskers). Each dot in the plot is an amino acid mutation (i.e., a clinical case report). Statistics: one-way ANOVA followed by Tukey’s post hoc test. In all cases, we used *n* = 171 (mild), *n* = 70 (moderate), and *n* = 123 (Severe). *** indicate Tukey’s post hoc *p* values < 0.001; **p value < 0.01; *p value < 0.05.

Therefore, we created a different representation of the FVIII protein structure and used machine learning (ML) methods to analyze all protein properties in conjunction. We named this framework Hema-Class (Fig. 1a). To uncover hidden patterns in the data, ML methods require large amounts of input information, but since HA is a rare disease, we did not have the amount of data usually employed by ML in biomedical applications. We solved this problem by establishing a systematic data curation strategy, and after training Hema-Class with a limited amount of data, we challenged it with prediction tasks of increasing difficulty to gradually fine-tune its parameters. Importantly, we designed Hema-Class as an open-source system that can be immediately retrained when new HA mutations appear in the literature.

Finally, we used Hema-Class to make predictions of the severity of all possible FVIII mutations—including those not yet reported in the medical literature. We grounded the reliability of these predictions in the close agreement between Hema-Class and in vitro mutagenesis assays, as well as hundreds of clinical reports of FVIII mutations.

In summary, the contributions of this study are twofold: first, we established an ML approach based on limited clinical and molecular data, and second, we used this system to advance the understanding of the FVIII protein by indicating which mutations are neutral or detrimental to its function. We anticipate that this study will enable other rare diseases to benefit from ML and will contribute to the engineering of better recombinant FVIII therapeutics.

## Results

### Properties of FVIII protein indicate differences in disease severity

As an essential part of the coagulation cascade, the FVIII protein works as a co-factor, binding activated coagulation factor IX (FIXa), coagulation factor X (FX), and the phospholipid membrane of platelets^1,2^. In this delicate interaction with multiple molecular partners, even single nucleotide changes can destabilize the FVIII protein structure and compromise its function. Therefore, we collected several properties of all amino acids of the FVIII structure^13^ (Protein Data Bank (PDB) accession 2R7E), including the surface exposure area, hydrophobicity and torsion angles of the amino acids, and searched for clinical reports that described HA cases caused by mutations at each of those residues. In addition, we verified that the properties of the structure we used in this study strongly correlated to the properties of another structure published recently^14^ (PDB accession 6MF2—Supplementary Fig. 1), thus, we did not use the 6MF2 structure for the analyses.

As it happens with rare diseases, the size of the input data often hampers its use for robust statistical analyses; we circumvented this issue by a careful and strict data sanitation strategy (“Methods”). We designed an input set with 443 non-synonymous mutations at 364 different FVIII positions (202 mild, 77 moderate, and 164 severe), maximizing the amount of information without biasing the dataset toward any particular variable (Fig. 1a).

Similar to previous studies^7–11^, we found that mutations at positions with low solvent-accessible and low solvent-excluded surface areas, as well as in highly conserved residues are related to more severe phenotypes (Fig. 1b and Supplementary Fig. 2). As it happens with other proteins^15,16^, this indicates that the substitution of conserved FVIII amino acids buried at the core of FVIII interferes with its conformation, function, and ability to bind other proteins^17–19^.

Next, we created a residue interaction network (RIN) of the FVIII protein. In this network, each amino acid corresponds to a *node*, and two nodes are connected by an *edge* if the amino acids are in close proximity to each other in the protein structure (Fig. 1a). This type of representation provides valuable information about the importance of amino acids and has been used to design new peptides and to understand the fundamental properties of proteins (reviewed extensively by others^20^).

In our RIN we found that the degree and the Burt’s constraint^21^ values are good indicators of the disease phenotype. These centrality measures quantify respectively the number of connections a node has, and how central or isolated a node is in the RIN (i.e., low constraint indicates a central amino acid and high constraint indicates a peripheral or isolated residue—Fig. 1b).

We found that mutations to amino acids that are central to the network (i.e., high degree and low constraint values) are related to more severe phenotypes. For instance, among the top 1% most connected amino acids in the RIN network, 34 out of 44 have reported mutations associated with HA (77%); on the other end, among the 1% least connected amino acids, only 21 out of 74 have cases reported (28%).

Residues Phe447 and Lys444 are among the most central residues in the FVIII RIN, and upon mutation to Ser and Arg/Thr/Asn, respectively, a severe form of HA ensues^22–24^. On the other hand, substitutions of amino acids that are peripheral in the residue network tend to cause mild or moderate symptoms. The mutations Ala394Ser, Thr454Ile, and Val578Ala in the A2 domain result in mild HA phenotypes^25,26^. A comprehensive characterization of the FVIII RIN is presented in a separate study (Lopes et al., submitted).

Taken together, our results indicate that in addition to the properties inferred directly from the protein structure, properties derived from the RIN offer a distinct perspective on the importance of the residues in FVIII and their relation to HA phenotypes.

### Development of an ML classifier for HA

Although the study of individual characteristics of the FVIII protein helps to understand the disease severity caused by mutations, these properties are all interrelated (e.g., more conserved residues usually have less surface exposure^27^). Therefore, we studied not only protein properties in isolation but how they jointly determine the severity of HA.

For this purpose, we used supervised ML classifiers. These algorithms receive as input the characteristics of the FVIII protein and a class label indicating the phenotype that a mutation causes. They output a score indicating the probability that a single point mutation will cause a severe HA phenotype (we named it the *Severity Score*) (Supplementary Table 1).

**Fig. 2:**
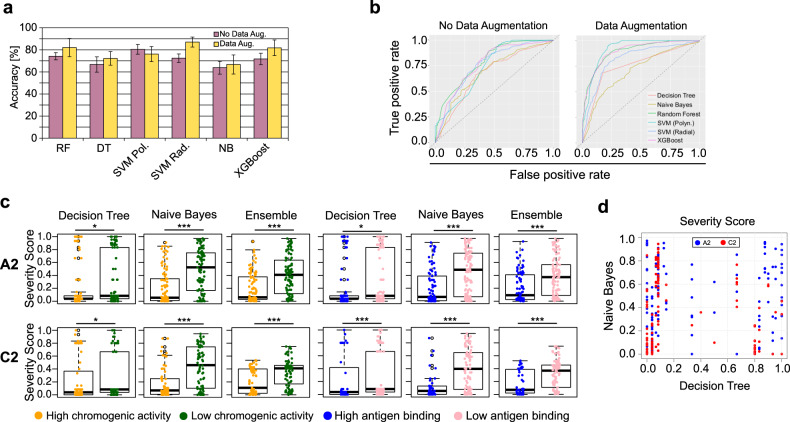
Machine-learning classifier properties. **a** Comparison of the accuracy of the different classifiers. These are the averages of 10-fold cross-validation for each classifier. The bars depict mean values and error bars, the standard deviation. **b** The AUC (Area Under the Receiver Operating Characteristic curve depicts the relation between the true positive and the false-positive rates. Points close to (0,1) indicate a better classification. The diagonal line represents a random classifier for a class-balanced dataset, i.e., any result below this line is worse than assigning labels randomly. **c** Distribution of the Severity Scores of two classifiers and an ensemble, and their relation to the in vitro chromogenic activity and the expression/secretion ability of the in vitro FVIII mutant constructs. In total, 344 alanine mutants were used (205 for A2 and 139 for C2). The boxplots depict the median (centerline), the first and third quartiles (lower- and upper-bounds), and 1.5 times the inter-quartile range (lower- and upper whiskers). Each dot in the plot is an amino acid mutation (i.e., an in vitro alanine mutant construct). **d** The Severity Score prediction of two classifiers for the chromogenic activity of FVIII mutants. The lack of correlation indicates that the classifiers are assigning different probabilities for the same instance—namely, having a different perspective about the real classification of mutants; this observation led us to combine their prediction values to come closer to the real activity of FVIII mutants. In all cases, we used the unpaired, two-sided Wilcoxon test (*** indicate *p* values < 0.001; **p value < 0.01; *p value < 0.05). SVM support vector machine, DT decision tree, NB naïve Bayes.

Using the maximally informative dataset that we created previously (443 instances, 164 severe, and 279 mild/moderate), and a cross-validation procedure (“Methods”), we compared six classification algorithms with and without data augmentation^28,29^. Overall, we obtained an accuracy of 66–87% in our classification (Fig. 2a, b), suggesting that a small but informative training set augmented by a reliable statistical technique contained enough information to effectively induce the classifiers to learn the hidden patterns of the data.

Next, we posed the first challenge to all classifiers: to predict experimental mutations on the FVIII protein that were not used during the training phase.

We used an in vitro mutagenesis screening where almost all of the residues of the A2 and the C2 domains were mutated to alanine^30,31^. The A2 domain is important for the FVIII activity because in addition to its role in stabilizing the protein structure^32^, it contains a binding site for FIXa (ref. ^33^). In a similar fashion, the C2 domain is critical because it contains hydrophobic residues involved in interactions both with the von Willebrand factor as well as with the phosphatidyl-l-serine containing membrane^34^. Importantly, due to the amino acid composition of these domains, the binding sites to coagulation factors also form epitopes targeted by the different inhibitor antibodies developed by patients during treatment^35,36^.

In those alanine mutation assays, 344 mutant FVIII constructs were expressed in COS-1 cells, and the products were subject to a chromogenic assay to measure their level of thrombin production. The ELISA assay was used to assess the efficiency of FVIII mutant constructs expression and secretion (often referred as antigen assay because they measure both functional and nonfunctional FVIII proteins by the amount of FVIII antigen (protein) immobilized on the ELISA plate. Two commercial kits were used to apply the “sandwich” method, where the antigen (FVIII construct) is captured between two layers of antibodies^30,31^.

We used the 6 algorithms to predict the effects of the alanine mutations on each residue and found that two of the algorithms successfully identified mutants that did not impair the production of thrombin and its secretion (>50% wild type) (Fig. 2c, d, Supplementary Fig. 3). In most cases, the mutants with lower chromogenic and secretion activities had significantly higher Severity Scores, indicating that the classifiers successfully learned the hidden properties of the clinical and molecular data and made inferences about unseen FVIII variants. Importantly, these results made clear that only considering the accuracy of a training/testing procedure would be misleading; thus, presenting the classifiers with unseen data was essential to select the best algorithms for the next steps.

Interestingly, we verified that the two classifiers that performed better in this challenge outputted different Severity Scores for the same instances (Fig. 2e). Thus, we leveraged on this difference and created a bagging-based ensemble of classifiers by averaging the Severity Scores of both classifiers—we named this ensemble the *Hema-Class*.

We found that Hema-Class predictions had less variance than either algorithm alone (Fig. 2c, d). As demonstrated in previous studies^37,38^, ensembles of classifiers closely approximate predictions to the real values of instances and avoid the biases that all algorithms inevitably have.

Along these lines, one aspect that we did not address in the present study are the so-called cross-reactive material (CRM+) mutations, where the mutant protein exhibits relatively high antigen binding, but low chromogenic activity (in clinical reports and in the in vitro data we observed less than 100 such cases). As more data becomes available, Hema-Class can be retrained using CRM+ and CRM− input sets separately.

Nevertheless, our results indicate that even with limited input data—as is often the case for rare diseases—it was possible to build a ML framework that had a close agreement with in vitro and clinical data. Finally, Hema-Class succeed in one of the critical tasks of ML, namely, to be trained using one type of input data (clinical and molecular), and to predict another (in vitro mutagenesis).

### Predicting the severity of all possible FVIII mutations

With the confidence that Hema-Class effectively generalized to unseen FVIII mutations, we decided to make predictions about the HA severity that ensues upon mutating all FVIII positions to all 19 remaining amino acids. The most up-to-date database contains point mutations in only ~730 positions (~30–50% of the FVIII residues, depending on the stage of its life cycle); therefore, predictions of unseen amino acid substitutions are relevant to understand which regions and mutations are detrimental to the FVIII activity.

We created a dataset with mutations in all of the wild-type FVIII positions to each of the remaining 19 amino acids. In total, this dataset had more than 25,000 instances—more than 50 times the size of our training set. Different from our previous analyses, the values of the properties derived from the 3D structure remained constant for the same amino acid, and only the distance between the wild-type residue and the new amino acid varied. For example, the instances with mutations Leu26Arg and Leu26Pro have the same surface exposure values, degree, and Burt’s constraint (of the leucine at position 26), the only difference between them is the distance between arginine and leucine, and proline to leucine (we describe the distance measure in the Methods section); this setting (i.e., varying only one feature while keeping the others constant), is a major challenge for ML classifiers because it tests the sensibility of the classifiers to small changes in a single variable.

We used Hema-Class to predict the Severity Score of 530 point-mutations described in the literature but previously unused in our study (Supplementary Table 2).

**Fig. 3:**
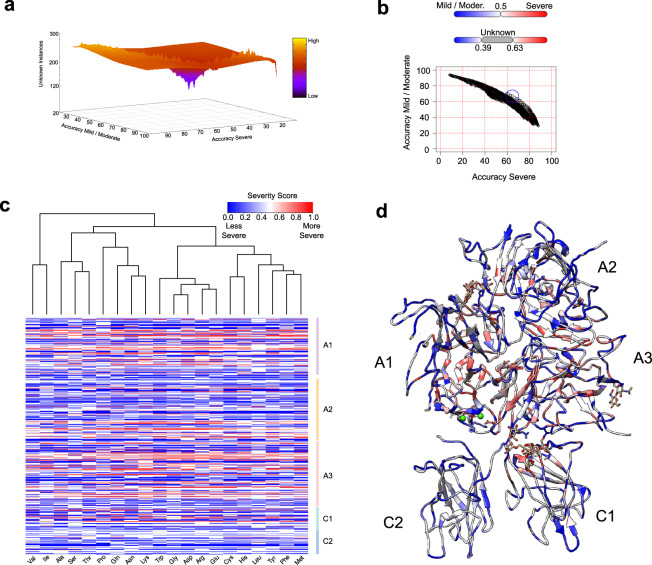
Hema-Class refinement and predictions. **a** The accuracy of the severe and the mild/moderate classes varies depending on the Severity Score cutoffs. Changing the Severity Score cutoffs effectively creates a “gray zone” where fewer instances are classified, but with higher accuracy. Depicted is the landscape created by varying the cutoff values. **b** Each dot is a combination of a minimum and maximum Severity Score cutoffs, and the blue circle depicts the values that enabled Hema-Class to achieve the best classification of FVIII point-mutations not seen during the training phase (~60% for both severe and mild/moderate classes). **c** Predicted Severity Score of all possible mutations of the FVIII amino acids in the FVIII structure (available in Supplementary Table 2). **d** FVIII structure colored by the Severity Score of each amino acid. Red indicates a higher probability of loss of function.

We opted to classify less instances, but with greater accuracy; therefore, we varied the Severity Score thresholds to create a “gray zone”, where instances were classified as *unknown* instead of receiving an incorrect classification (Fig. 3a). After close inspection of the Severity Score and the accuracy behavior, we defined that instances with Severity Scores in the interval [0.39–0.63) would be classified as “unknown” (161 instances).

With these thresholds, Hema-Class yielded an overall accuracy of 62% (Fig. 3b). We confirmed these results predicting the severity of ~1000 reported mutations of the CHAMP database (Methods—Supplementary Fig. 4), indicating that although the correlation is not perfect, severe HA phenotypes are consistently associated with higher Severity Scores.

Next, these results enabled us to predict the Severity Score of previously unreported ~17,500 FVIII mutations.

We found that ~4000 mutations had high Severity Scores, and Fig. 3b shows that there are regions of the FVIII protein that are more prone to cause severe HA symptoms if mutated. For instance, while most regions of the C1 and C2 domains might tolerate different substitutions, the buried regions of the A1 and A3 domains are more likely to cause severe HA symptoms independently of the amino acid replacement (Fig. 3c, all predictions are listed in Supplementary Table 2). Interestingly, we found that Thr68, Leu117, Asp186, Met339, Trp1854, and Gly1942 are the most sensitive positions and received high Severity Scores for most amino acid substitutions. In accordance with previous studies, our results indicate that the FVIII protein core is composed of a complex network of inter-atomic interactions, and mutations leading to disruption of this intricate architecture leads to the loss of FVIII activity^9–11,39^.

Taken together, our results demonstrate that it was possible to train Hema-Class using a small input set, and after confirming its agreement with in vitro and clinical data, we were confident to make predictions about unseen hypothetical mutations. The ability to adjust the Severity Score thresholds added flexibility to the framework and created a trade-off between a smaller accuracy and more instances classified, or fewer instances classified but with more certainty.

## Discussion

In this study, we analyzed single amino acid mutations to identify the properties of the FVIII protein structure that are strong indicators of HA severity. After proper data sanitation, we obtained a compact yet informative dataset of HA characteristics and used it as input to an ML classifier (the Hema-Class framework, Fig. 1a). After verifying the agreement of our predictions to in vitro and clinical reports, the Hema-Class was subsequently used to make predictions about the severity of HA for all possible FVIII non-synonymous mutations—including those not yet reported in the scientific literature.

In its activated form, the FVIII protein interacts with FIXa, FX, and the platelet’s phospholipid membrane. These transient and precise interactions can be disturbed by even one amino acid substitution—and the degree of disruption depends on the position and the type of amino acid replacement^27^. Similar to previous studies, we verified that the surface exposure and the conservation of a residue have a strong relation to the HA phenotype^7–12^. Notorious examples are mutations at residues Asp186 and Met339, buried inside FVIII and whose side-chains are involved in a complex hydrogen-bond network and contribute with major energy in copper binding^26,40^.

Next, we created a representation of the FVIII protein structure (RIN), and with this convenient and intuitive representation we visualized and quantified the “centrality” of all residues in the structure; we present a complete characterization of this network in a separate study (Lopes et al., submitted). We verified that substitutions to the critical residues of the FVIII proteins (i.e., the most centrally connected ones) caused detrimental effects on the FVIII function (Fig. 1b). In general, the most connected residues were those buried at the core of the A1 and A3 domains that kept the FVIII structure stable^13,41^. In most proteins, substitutions of the highly conserved hydrophobic residues (usually at the core), lead to disruption of the protein structure due to side-chain clashes and separation of hydrogen and non-covalent bonds^27^. In the protein world conformation equals function, and it is a direct consequence that substitutions at these sites impair the FVIII activity.

With the structural properties collected and evaluated, the next step was to use them in conjunction to make predictions about HA severity—and for this purpose, we built a small yet highly informative input dataset for our ML framework (the Hema-Class).

After proper training and validation, we used Hema-Class to predict the severity of 344 alanine mutations in the A2 and C2 domains. We verified a close agreement between the in silico and in vitro results, as evidenced by the fact that the most dramatic reductions in the chromogenic and secretion activities of the FVIII protein were accompanied by high Severity Scores (Fig. 2c, d). For instance, Lys444Ala and Phe447Ala in the A2 domain (Severity Scores 0.97 and 0.91, respectively) had 0% of the wild-type chromogenic activity^31^. These encouraging results gave us confidence that Hema-Class captured FVIII properties that were previously only observable using in vitro assays.

Finally, the Hema-Class framework enabled us to make predictions of all possible mutations in the FVIII protein. Predicting more instances than are used during training is a notorious problem in ML^42^, but we addressed this issue using point-mutation data not used during the training phase to estimate the most appropriate Severity Score thresholds. With the data available at present, we achieved an accuracy of 62% (Fig. 3a) and identified the “hot-spots” of the FVIII protein at the core regions of the A1 and A3 domains (Fig. 3b, c). Supported by hundreds of clinical reports, we are confident that changes to these residues are detrimental to the FVIII function.

The agreement between Hema-Class predictions and the in vitro and clinical reports is not perfect. These discrepancies exist because clinical reports often have conflicting information (e.g., lack of agreement between chromogenic activity and clinical symptoms), and contacting the authors is infeasible, given that some studies were published decades ago. In addition, there are different methods to measure the FVIII co-factor activity (e.g., one-stage aPTT clotting assays and chromogenic substrate assay), and although their results agree in most cases^43^, these methods sometimes have discrepancies that induce the incorrect diagnostic and consequently affect the quality of the results outputted by Hema-Class. Moreover, the in vitro assays are not completely realistic because they lack components that are present in the human circulation (e.g., activated platelets, other host proteins, and the blood flow). Additionally, due to technical constraints at the time, the 2R7E structure^13^ was determined at a low-resolution (3.7 Å) and have regions that were not modeled or contain unfavorable torsion angles (Supplementary Fig. 5); for this reason, although the results presented here pave the way for the in silico prediction of disease severity, they should be interpreted with care in the light of these limitations.

Nevertheless, the Hemophilia community is actively working on these issues, and even with these adversities the Hema-Class framework uncovered meaningful patterns from clinical and molecular data; importantly, we designed the Hema-Class architecture in a way that it can be easily retrained as new data becomes available (see “Data availability”).

In summary, we have established a ML framework to study and anticipate the severity of HA-based on the characteristics of the FVIII protein structure. The incremental difficulty of the challenges presented to Hema-Class posed a formidable challenge to its capacity and gave us the confidence in its predictions. We believe that the challenge of designing a recombinant FVIII with a longer half-life and better immunogenic profile remains; however, being able to mechanistically study mutations in silico brings us a step closer to this goal.

## Methods

### Protein structure and its properties

We used the FVIII protein structure deposited in the PDB with the accession 2R7E (ref. ^13^). We extracted the structural properties from this structure using Chimera version 1.14 (ref. ^44^). The measures obtained using Chimera were areaSAS, areaSES, kdHydrophobicity, PSI, PHI, and the bFactor (Supplementary Table 1).

Next, we transformed this protein in an undirected, unweighted graph using RINerator version 0.5.1 (ref. ^45^) with the default parameters. We considered that two residues interacted if there was at least one edge between them, independently of the edge type. To analyze the RIN, we used R version 3.6.3 (https://www.R-project.org/) and the iGraph package, version 1.2.5 (ref. ^46^). With the iGraph package, we used the function *simplify* to remove redundant edges and self-interactions. Next, we calculated the degree, betweenness, closeness, Burt’s constraint^21^, Authority Score, Page Rank-like, KCore, and the Authority Score measures.

We obtained the conservation score from the ConsurfDB webserver^47^ and created the Ramachandran plot using a webserver from Anderson et al.^48^.

### Database sanitation

We manually queried the EAHAD database^49^ on 25th June 2020. We selected “Point” and “Polymorphism” (on type), and “Missense” (on variant effect) on the advanced search. It returned a total of 6051 rows. Next, we removed mutations on the signal peptide regions, or outside the mature form of the protein, as well as instances with 1-st/2-st FVIII:C > 100. We also removed non-numerical values on the FVIII:C column, substituted the values >5 for 5, <10 for 10, <11 for 11, “<1” or “<1” for “0”. We also removed instances with FVIII:C values that would lead to ambiguous diagnostics (e.g., “0 to 2”, “<1 to 2”, <2, etc).

We substituted FVIII:C that contained ranges (e.g., “10–24”) to the average value (in this example, 17). We removed instances without FVIII:C and without inhibitor information, and instances with discrepancies between “FVIII:C% (presumed 1-st)*” and “FVIII:C% (2-st/Chr)”, and one mutation encoding a stop-codon.

Finally, we removed instances with ambiguously reported classifications (e.g., “mild/moderate”, or “moderate/severe”).

For the ML validation step, we downloaded the CHAMP FVIII database (https://www.cdc.gov/ncbddd/hemophilia/champs.html), and considered only non-synonymous substitutions at single nucleotides, removed instances with ambiguously reported severities (e.g., “mild/moderate”, or “moderate/severe”), removed duplicated instances as well as mutations that would lead to stop codons, as well as mutations outside the coding regions. After this data sanitations step, ~1000 unique reported mutations were left.

### Amino acid distance index

We used the R package seqinR (ref. ^50^) to obtain 544 numerical properties for each amino acid. Next, we used the package AMAP (ref. ^51^) to perform a principal component analysis of this set, and reduced the number of properties to 19 components, while retaining 99% of the information in the dataset. We calculated the Euclidean distance between all amino acids, considering all 19 component values. This gave us a 20 × 20 matrix which was the distance index used in our analyses (Supplementary Table 1).

### Classifier methodology

Supervised learning is a subarea of ML focused on producing the best possible mapping (model) *f*:*χ*→Ƴ of examples *x*_*i*_ in some input space *χ* to class labels *y*_*i*_ in the output space Ƴ (ref. ^52^). In the context of this work, input examples are composed of protein features and class labels are the HA severities. Here we describe the mapping strategy adopted by the ML algorithms that we used.

### Experimental setup

The experimental setup designed to create the Hema-Class followed the following steps: preprocessing, training, and testing. Besides the preprocessing tasks already mentioned in the Results section, we also normalized all attributes to make sure our framework is not biased by data scales. We also removed all examples where values in at least one attribute was missing. We employed the tenfold cross-validation method to reduce the chances of estimating overfitted models, and to ensure that the same sets of examples were considered by the different ML algorithms. This enabled a fair training and testing for all algorithms. Finally, before proceeding with model training, we used a strategy of data augmentation to balance the dataset classes, given it was composed of examples of the classes “Severe” (*n* = 152) and “Mild/Moderate” (*n* = 263). The Adaptive Synthetic Sampling Approach for Imbalanced Learning (ADASYN)^29^ was used to balance the dataset by employing a weighted distribution for different minority class examples according to their level of difficulty in learning. ADASYN yields more synthetic data for the minority class examples that were harder to be predicted, thus improving the learning process by reducing the bias introduced by the class imbalance, and adaptively shifting the classification decision boundary towards the more complex examples. After using ADASYN, our augmented dataset contained 296 examples of the Severe and 263 of Mild/Moderate classes.

The training and test steps were performed using a grid search strategy to look for the best parametrization for all ML methods. To model the dataset using Decision Tree, we varied the minimum number of observations in a node before splitting the data within the interval minsplit = [2, 50]. The minimum number of observations (minbucket) in a terminal node (leaf) was searched in the interval [1, 20]. Finally, the complexity parameter (cp) lied in range [0.0001, 1].

The Random Forest (RF)^28^ was trained varying three parameters: a number of trees (ntree) in the interval [4,100]; the number of variables randomly sampled as candidates at each split (mtry) in the interval [2, 7]; and minimum size of terminal nodes or leaves (nodesize) between [1, 5].

The Support Vector Machine was assessed using the two best kernels according to a first empirical set of experiments: radial ${\it{ \in }}^{( - y[x - \omega ]^2)}$, and polynomial (*yω*'x+*c*)^*d*^, given *ω*,x is two position vectors representing examples. For the radial kernel, we analyzed the following parameters *y* = {0.01, 0.02, …, 1.5}, while the polynomial kernel was assessed using *c* = {0.1, 0.15, …, 2}, c = {0.1, 0.15, …, 2}, *d* = {2, 3, …, 5}.

The model obtained with Naïve Bayes has no parameter estimation.

Finally, the XGBoost method^53^ was estimated by running a grid search on the following parameters: maximum depth of a tree in {1, …, 25}, *y* is the L_2_ regularization (Ridge Regression) term on weights in the range [0, 1] to define the number of samples taken into consideration, *ɳ*∈[0,1] defining the learning rate by scaling the contribution of each tree, and obj is the loss function.

The best models obtained during the training phase with the tenfold cross-validation strategy were chosen by their relative performances in terms of the Kappa index and the Area Under the ROC Curve. The Kappa index measures the agreement between the predicted and expected values, thus emphasizing that the results were not obtained by chance. This coefficient subtracts the expected from the observed agreement to quantify the probability of correct classifications by chance^54^.

### Reporting summary

Further information on research design is available in the Nature Research Reporting Summary linked to this article.

## Supplementary information

Supplementary Information

Supplementary Data 1

Supplementary Data 2

Reporting Summary

## Data Availability

The datasets used in this study are available in the Supplementary Tables as well as in https://github.com/ricardoarios/hemophilia-A-FVIII-ML.

## Code availability

The source code used in this study is available at https://github.com/ricardoarios/hemophilia-A-FVIII-ML.
