# Supplementary material for: Prediction of hemophilia A severity using a small-input machine-learning framework
Source: NPJ Syst Biol Appl. 2021 May 25;7:22. doi: 10.1038/s41540-021-00183-9 (PMC8149871; doi:10.1038/s41540-021-00183-9)
Supplement: Supplementary file 1 — Supplementary Information [file 41540_2021_183_MOESM1_ESM.pdf]

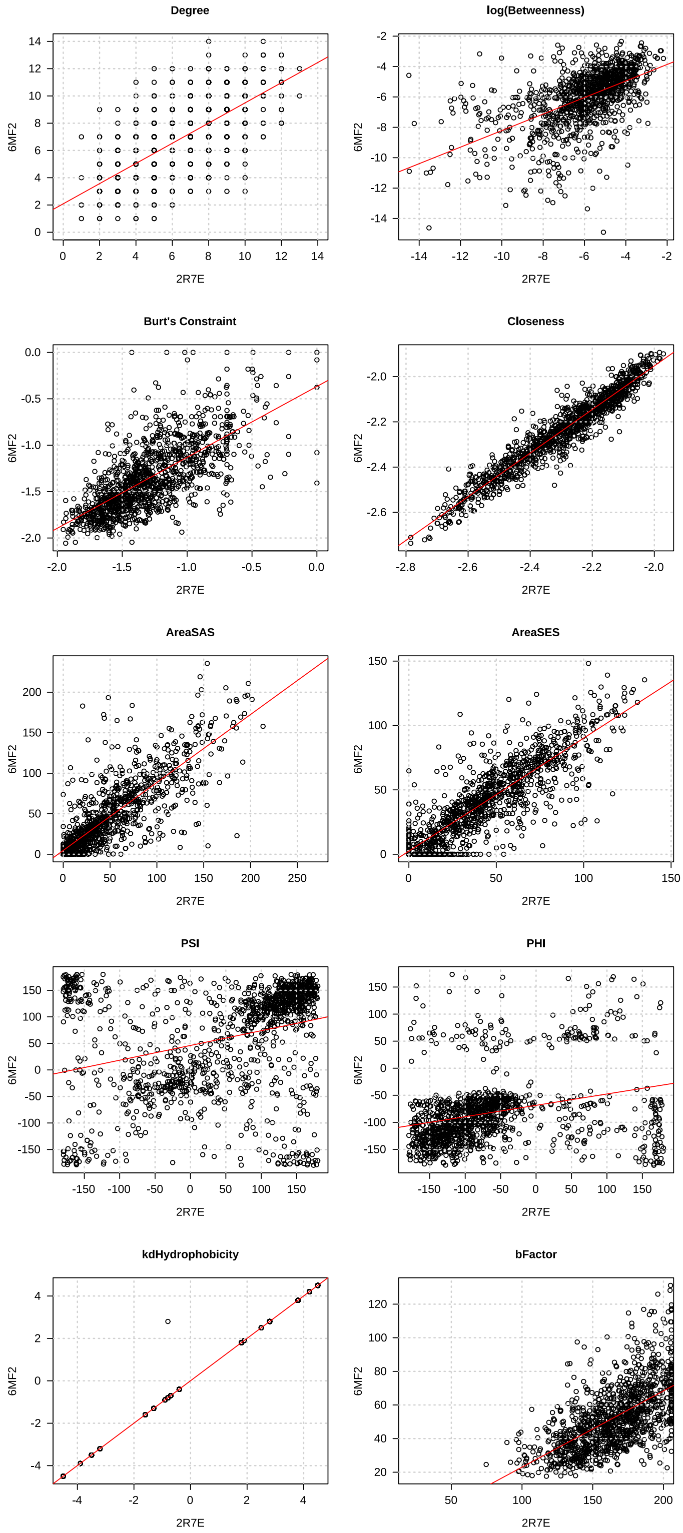

Supplementary Figure 1: Close resemblance of the structural properties derived from the 6MF2 and the 2R7E structure.

The properties of both protein structures were obtained using the methods described in the Methods section.

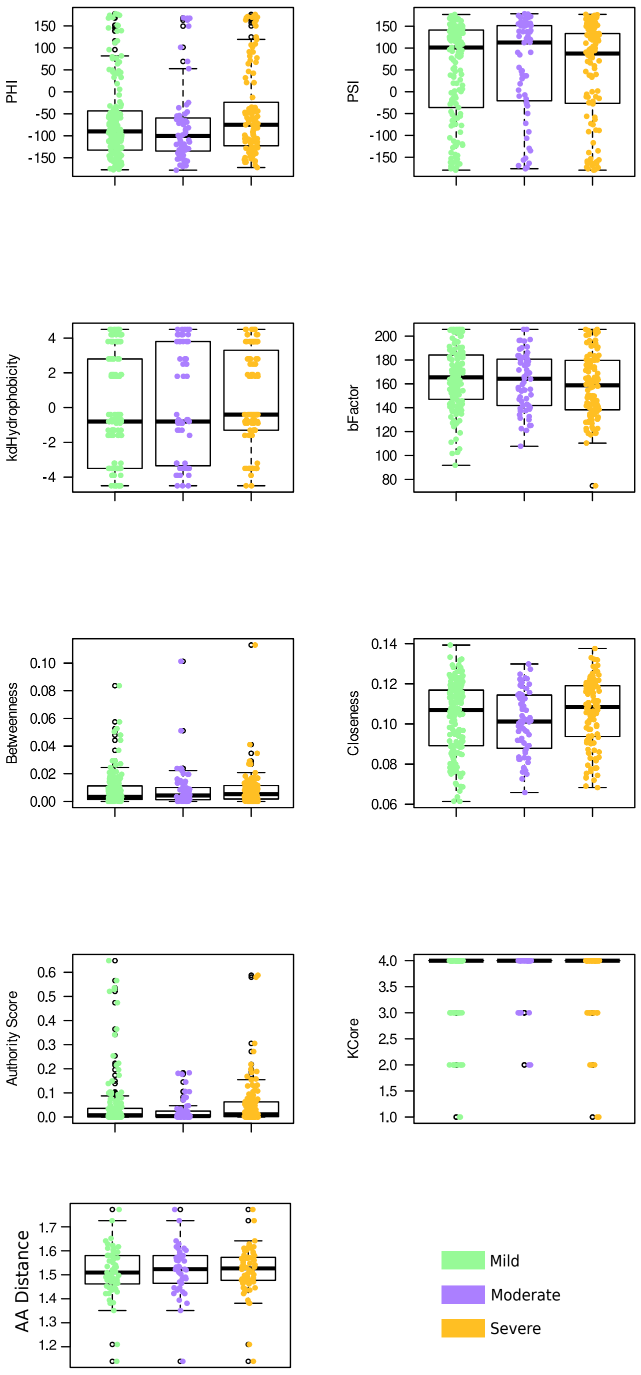

Supplementary Figure 2: Other structural properties and centrality measures.

We attempted to identify associations between structural properties of the FVIII protein structure (i.e., PHI, PSI, bFactor, kdHydrophobicity), and the severity of HA. We also tested the significance of the centrality measures of the RIN and the severity of the disease. In both cases we did not find significant differences. Statistics: One-way ANOVA followed by Tukey's Post-hoc test. In all cases, we used n=171 (Mild), n=70 (Moderate), and n=123 (Severe). *** indicate Tukey's Post-hoc p-values < 0.001; ** p-value < 0.01; * p-value < 0.05.
The boxplots depict the median (center line), the first and third quartiles (lower- and upper-bounds), and 1.5 times the inter-quartile range (lower- and upper whiskers). Each dot in the plot is an amino acid mutation (i.e., a clinical case report).

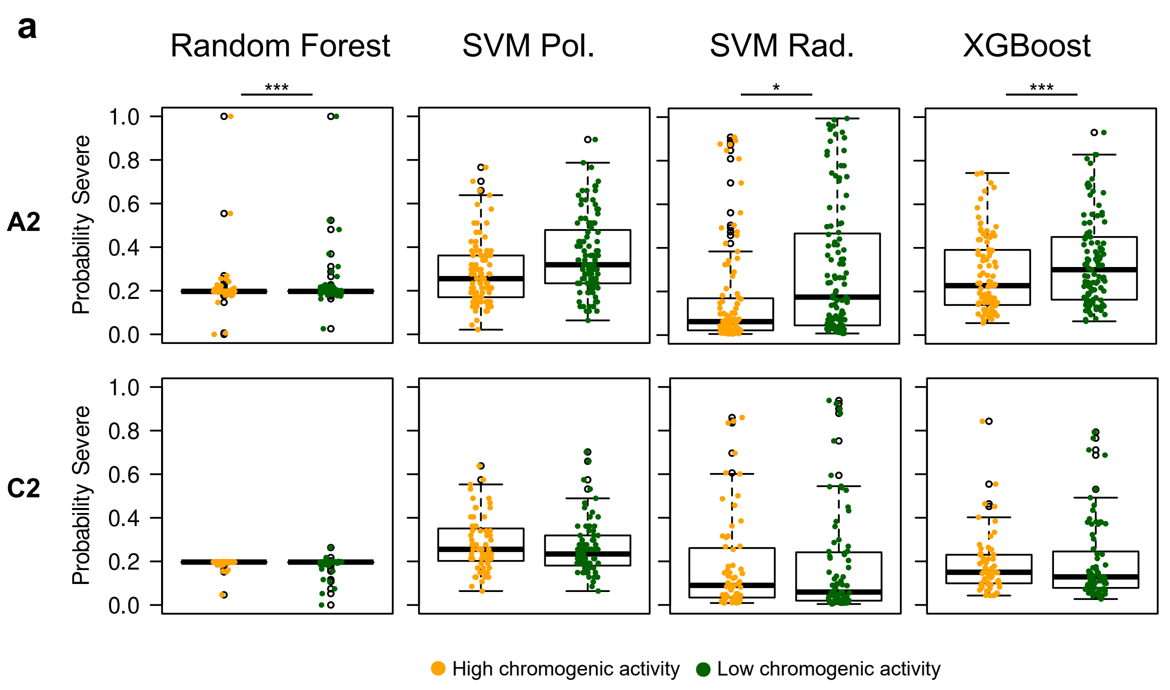

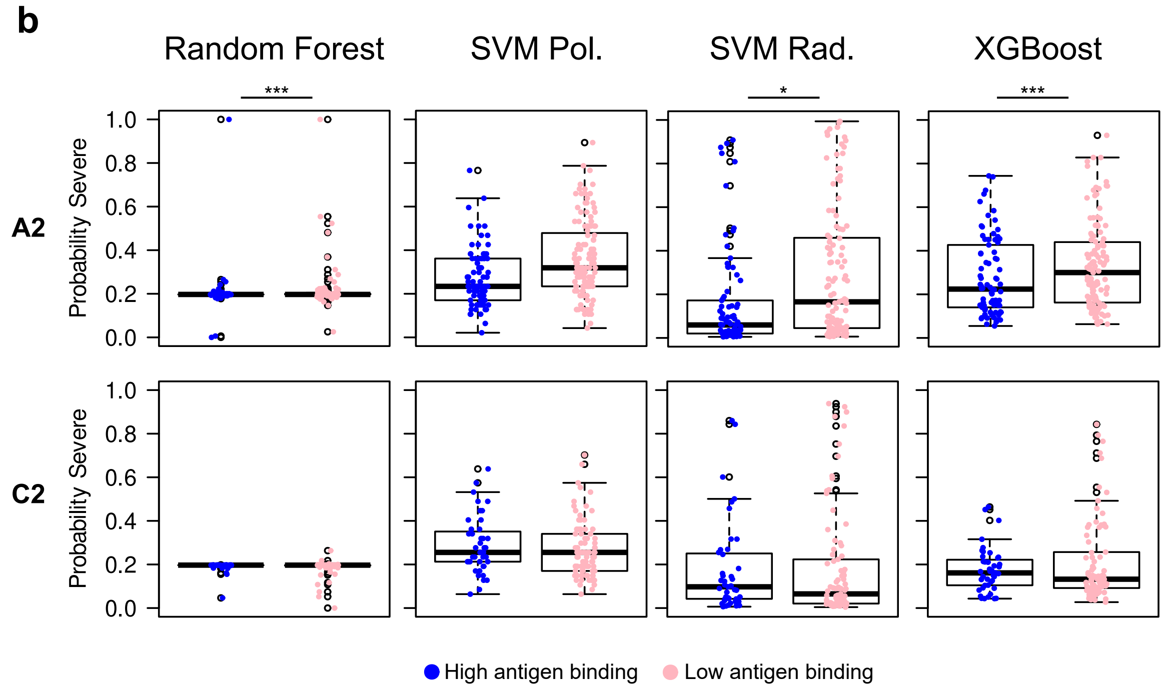

Supplementary Figure 3: Classifier comparison of the in vitro mutagenesis data.

a Distribution of the Severity Scores of four classifiers and their relation to the in vitro chromogenic activity of two domains of FVIII (A2 and C2). In total, 344 alanine mutants were considered (205 for the A2 and 139 for the C2 domains). **b** The same comparison for the expression/secretion binding ability of the in vitro mutants. In all cases, we used the unpaired, two-sided Wilcoxon test
(*** indicate p-values < 0.001; ** p-value < 0.01; * p-value < 0.05).
The boxplots depict the median (center line), the first and third quartiles (lower- and upper-bounds), and 1.5 times the inter-quartile range (lower- and upper whiskers). Each dot in the plot is an amino acid mutation (i.e., an *in vitro* alanine mutant construct).

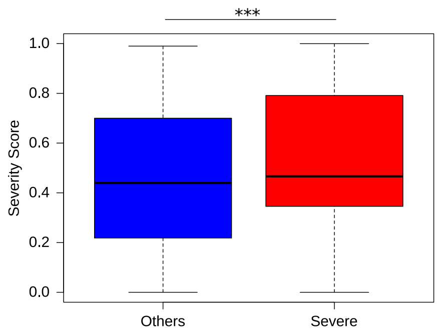

Supplementary Figure 4: Severity Score from mutations of the CHAMP database. In total, 1,015 reported mutations were considered after proper data sanitation (Methods). We used the unpaired, two-sided Wilcoxon test (*** indicates p-values < 0.001). The boxplots depict the median (center line), the first and third quartiles (lower- and upper-bounds), and 1.5 times the inter-quartile range (lower- and upper whiskers). We did not use dotplots because the excessive number of points would obscure the image.

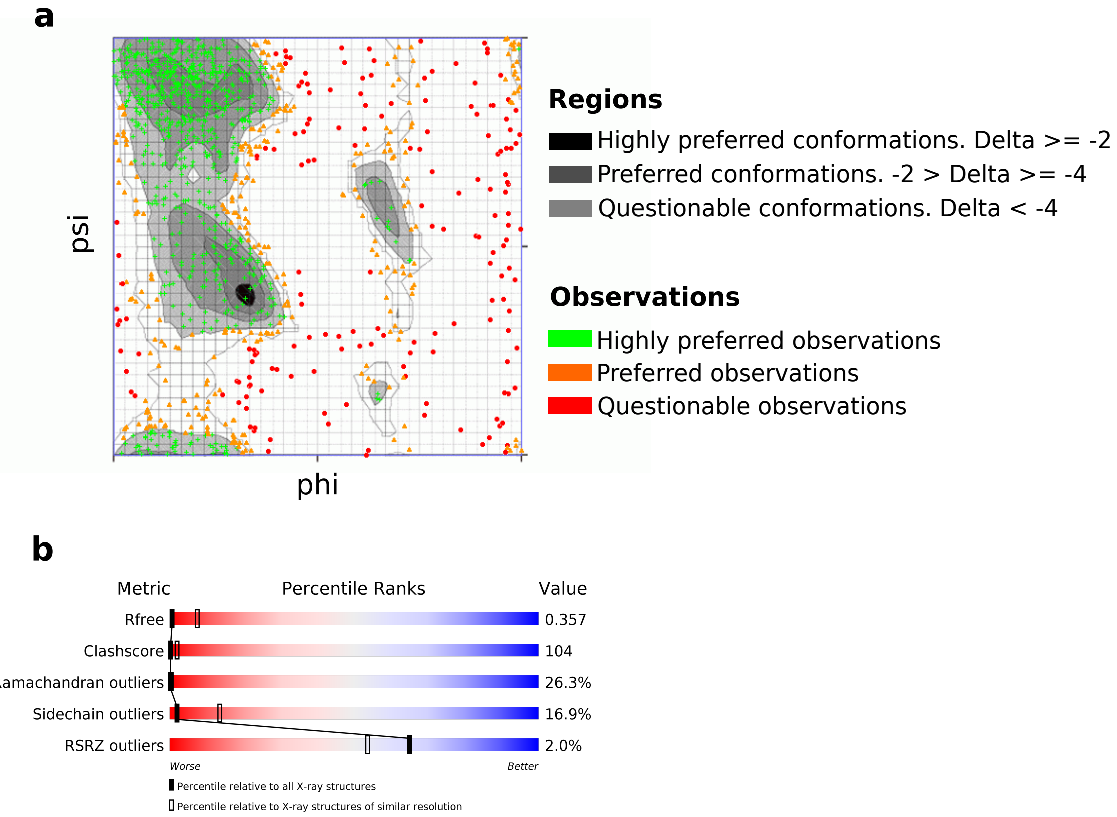

Supplementary Figure 5: Quality measures of the FVIII structure.
a Depicted is the Ramachadran plot of the 2R7E structure (PDB ID 2R7E). Here, highly preferred observations (green) are 730 amino acids (61.86%), the preferred observations (orange) are 280 amino acids (23.72%), and questionable observations (red) are 170 residues (14.40%).
b Different quality measures of the structure, obtained from the PDB website. While most measures at on the lower side, they are comparable to other ~1,000 structures determined at similar resolution (3.7 Å).

Supplementary Tables

Supplementary Table 1: Properties and centrality measures of the FVIII protein structure.

This table contains the data of the 443 residues of the FVIII protein structure (PDB ID 2R7E that we used for training the Hema-Class. It also contains the protein properties of all residues of the FVIII protein structure; it can be used to study and to retrain the Hema-Class when new clinical reports with novel mutations become available.

Supplementary Table 2: Predicted severity of all-to-all FVIII amino acid mutations.

This table contains the predicted Severity Score outputted by Hema-Class for all residues changing from the wild-type amino acids to all other 19 amino acids. The instances used for training were not included in this table. Additionally, it also lists the agreement of our predictions to 530 clinical reports derived from the EAHAD database.
