# Supplementary material for: Prediction of hemophilia A severity using a small-input machine-learning framework
Source: NPJ Syst Biol Appl. 2021 May 25;7:22. doi: 10.1038/s41540-021-00183-9 (PMC8149871; doi:10.1038/s41540-021-00183-9)
Supplement: Supplementary file 4 — Reporting Summary [file 41540_2021_183_MOESM4_ESM.pdf]

nature research

Corresponding author(s): *Double-blind peer review submissions: write DBPR and your manuscript number here instead of author names.*

Last updated by author(s): *YYYY-MM-DD*

# Reporting Summary

Nature Research wishes to improve the reproducibility of the work that we publish. This form provides structure for consistency and transparency in reporting. For further information on Nature Research policies, see our Editorial Policies and the Editorial Policy Checklist.

## Statistics

For all statistical analyses, confirm that the following items are present in the figure legend, table legend, main text, or Methods section.

| n/a | Confirmed | |
|---|---|---|
| ☐ | ☒ | The exact sample size (*n*) for each experimental group/condition, given as a discrete number and unit of measurement |
| ☐ | ☒ | A statement on whether measurements were taken from distinct samples or whether the same sample was measured repeatedly |
| ☐ | ☒ | The statistical test(s) used AND whether they are one- or two-sided<br>*Only common tests should be described solely by name; describe more complex techniques in the Methods section.* |
| ☐ | ☒ | A description of all covariates tested |
| ☐ | ☒ | A description of any assumptions or corrections, such as tests of normality and adjustment for multiple comparisons |
| ☐ | ☒ | A full description of the statistical parameters including central tendency (e.g. means) or other basic estimates (e.g. regression coefficient) AND variation (e.g. standard deviation) or associated estimates of uncertainty (e.g. confidence intervals) |
| ☐ | ☒ | For null hypothesis testing, the test statistic (e.g. *F*, *t*, *r*) with confidence intervals, effect sizes, degrees of freedom and *P* value noted<br>*Give P values as exact values whenever suitable.* |
| ☒ | ☐ | For Bayesian analysis, information on the choice of priors and Markov chain Monte Carlo settings |
| ☒ | ☐ | For hierarchical and complex designs, identification of the appropriate level for tests and full reporting of outcomes |
| ☒ | ☐ | Estimates of effect sizes (e.g. Cohen's *d*, Pearson's *r*), indicating how they were calculated |

*Our web collection on statistics for biologists contains articles on many of the points above.*

## Software and code

Policy information about availability of computer code

| | |
|---|---|
| Data collection | The data was collected from public databases, including the EAHAD FVIII, and CHAMPS F8 mutation databases. The software used was R statistical package and its machine learning package (MLR). Protein analysis was performed with Chimera. |
| Data analysis | We used these programs as well as our in-house scripts for all analyses reported in this study. All source code and datasets are available at https://github.com/ricardoarios/hemophilia-A-FVIII-ML |

For manuscripts utilizing custom algorithms or software that are central to the research but not yet described in published literature, software must be made available to editors and reviewers. We strongly encourage code deposition in a community repository (e.g. GitHub). See the Nature Research guidelines for submitting code & software for further information.

## Data

Policy information about availability of data

All manuscripts must include a data availability statement. This statement should provide the following information, where applicable:

- Accession codes, unique identifiers, or web links for publicly available datasets
- A list of figures that have associated raw data
- A description of any restrictions on data availability

The datasets and source code used in this study are available at https://github.com/ricardoarios/hemophilia-A-FVIII-ML.

# Field-specific reporting

Please select the one below that is the best fit for your research. If you are not sure, read the appropriate sections before making your selection.

☒ Life sciences ☐ Behavioural & social sciences ☐ Ecological, evolutionary & environmental sciences

For a reference copy of the document with all sections, see nature.com/documents/nr-reporting-summary-flat.pdf

# Life sciences study design

All studies must disclose on these points even when the disclosure is negative.

| | |
|---|---|
| Sample size | No sample size was explicitly performed. We used the algorithm SMOTE for data augmentation (see Methods). |
| Data exclusions | *Describe any data exclusions. If no data were excluded from the analyses, state so OR if data were excluded, describe the exclusions and the rationale behind them, indicating whether exclusion criteria were pre-established.* |
| Replication | We manually queried the EAHAD database48 on 25th June 2020. We selected 'Point' and 'Polymorphism' (on type), and 'Missense' (on variant effect) on the advanced search. It returned a total of 6,051 rows. Next, we removed mutations on the signal peptide regions, or outside the mature form of the protein, as well as instances with 1-st/2-st FVIII:C > 100. We also removed non-numerical values on the FVIII:C column, substituted the values >5 for 5, <10 for 10, <11 for 11, "<1" or "< 1" for "0". We also removed instances with FVIII:C values that would lead to ambiguous diagnostics (e.g., "0 to 2", "<1 to 2", <2, etc).<br>We substituted FVIII:C that contained ranges (e.g., "10 to 24") to the average value (in this example, 17). We removed instances without FVIII:C and without inhibitor information, and instances with discrepancies between "FVIII:C% (presumed 1-st)*" and "FVIII:C% (2-st/Chr)", and one mutation encoding a stop-codon.<br>Finally, we removed instances with ambiguous reported classifications (e.g., "mild/moderate", or "moderate/severe"). |
| Randomization | No randomization was performed. |
| Blinding | Blinding was not relevant to this study. |

# Reporting for specific materials, systems and methods

We require information from authors about some types of materials, experimental systems and methods used in many studies. Here, indicate whether each material, system or method listed is relevant to your study. If you are not sure if a list item applies to your research, read the appropriate section before selecting a response.

## Materials & experimental systems

| n/a | Involved in the study | |
|---|---|---|
| ☒ | ☐ | Antibodies |
| ☒ | ☐ | Eukaryotic cell lines |
| ☒ | ☐ | Palaeontology and archaeology |
| ☒ | ☐ | Animals and other organisms |
| ☒ | ☐ | Human research participants |
| ☒ | ☐ | Clinical data |
| ☒ | ☐ | Dual use research of concern |

## Methods

| n/a | Involved in the study | |
|---|---|---|
| ☒ | ☐ | ChIP-seq |
| ☒ | ☐ | Flow cytometry |
| ☒ | ☐ | MRI-based neuroimaging |
